# Synchrony can destabilize reward-sensitive networks

**DOI:** 10.3389/fncir.2014.00044

**Published:** 2014-04-30

**Authors:** Michael Chary, Ehud Kaplan

**Affiliations:** Department of Neuroscience, Icahn School of Medicine Mount Sinai, Friedman Brain InstituteNew York, NY, USA

**Keywords:** cortical networks and systems, synchrony code, computational models in psychiatry, plasticity and learning, substance abuse

## Abstract

When exposed to rewarding stimuli, only some animals develop persistent craving. Others are resilient and do not. How the activity of neural populations relates to the development of persistent craving behavior is not fully understood. Previous computational studies suggest that synchrony helps a network embed certain patterns of activity, although the role of synchrony in reward-dependent learning has been less studied. Increased synchrony has been reported as a marker for both susceptibility and resilience to developing persistent craving. Here we use computational simulations to study the effect of reward salience on the ability of synchronous input to embed a new pattern of activity into a neural population. Our main finding is that weak stimulus-reward correlations can facilitate the short-term repetition of a pattern of neural activity, while blocking long-term embedding of that pattern. Interestingly, synchrony did not have this dual effect on all patterns, which suggests that synchrony is more effective at embedding some patterns of activity than others. Our results demonstrate that synchrony can have opposing effects in networks sensitive to the correlation structure of their inputs, in this case the correlation between stimulus and reward. This work contributes to an understanding of the interplay between synchrony and reward-dependent plasticity.

## 1. Introduction

Synchrony refers to a coordinated pattern of network activity. Synchrony occurs between (i) action potentials, (ii) local field potentials, or (iii) action potentials and local field potentials. The latter two types of synchrony are frequently called coherence. Neural networks with strong recurrent connections can demonstrate synchronous activity that persists over seconds to minutes (Tetzlaff et al., 2012). Changing synaptic strengths allows that activity to persist over longer time scales (Holtmaat and Svoboda, 2009).

Synchrony between action potentials helps localize sounds (Joris et al., 1998), signal the direction of motion (Meister et al., 1995; Meister and Berry, 1999), and discriminate among odors (Stopfer et al., 1997; Tetzlaff et al., 2012).

When exposed to addictive substances, only some individuals develop an addiction or dependence (Ersche et al., 2010). Of those who become addicted or dependent, only some respond to treatment (Gawin, 1991). Alterations in activity-dependent learning in areas of the brain involved in reward processing are important in the pathogenesis of addictive disorders (Koob and Le Moal, 2005). Increased synchrony can predict intoxication (Li et al., 2011), resilience, susceptibility (Coullaut-Valera et al., 2014), or likelihood of relapse (Camchong et al., 2013), depending on in which brain region the synchrony manifests.

These observations suggest that many aspects of addiction can be understood as changes in the structure of synchronization of neural networks. To explore this, we study the stability of a pattern of activity in the face of different stimulus-reward inputs.

## 2. Results

### 2.1. Summary of model

Equation (1) describes the dynamics of a group of neurons, **v**. Those neurons interact linearly with each other according to the intrinsic connection matrix **M**, and receive input, **u**, weighted according to the feedforward connection matrix **W**. The weights in **W** depend on (i) the correlation between the stimulus, **u**, and network activity, **v**, denoted **u** ⊗ **v**, and (ii) the correlation between the the stimulus, **u**, and the reward associated with the stimulus, **r**, denoted **r** ⊗ **v**. The second line in Equation (1) is a linear differential equation in **M**, which means that it can only remove pairwise correlations.

The top line of Equation (1) describes the firing rate of a population of neurons. That firing rate decays in the absence of recurrent or feedforward input. The second line implements Hebbian modification of the feedforward weights, modulated the by the reward associated with the stimulus, **r**. The third line implements anti-Hebbian modification of the recurrent weights. Anti-Hebbian modification prevents the network from responding identically to inputs with the same amount of active units.

(1) $$\begin{array}{l} \;\tau_{v}\frac{dv}{dt}=-v+M\;\cdot \;\left(\text{tanh }v\right)+W\;\cdot \;u\\ \tau_{W}\frac{dW}{dt}=K\;\cdot \;W\;\cdot \;u\left(r-v\right)\\ \tau_{M}\frac{dM}{dt}=\left(I-M\right)-\left(W\;\cdot \;u\right)v \end{array}$$

The importance of correlations arises directly from the bottom two lines in Equation (1) because the outer product of two vectors can be interpreted as the cross-correlation between those two vectors. In this paper, we only consider 1-dimensional stimuli for simplicity. The dependence of the dynamics of connections among neurons on the correlation between stimulus activity and network activity allows patterns of network activity that are very far from **v**_∞_ to maintain stable connections between neurons.

Connections between units in the network stabilize, that is $\frac{d}{dt}M\to 0$, when the correlation between network activity, **v**, and the filtered version of the input, **W** · **u**, lies parallel to the deviation between the connection matrix, **M** and the identity matrix, **I**. Connections between the network and input stabilize, that is $\frac{d}{dt}W\to 0$, when network activity accurately predicts the reward, **r** = **v** or the neurons in the network become autonomous, **M** = **I** so **K** = **0**.

### 2.2. Computational results

#### 2.2.1. Stimuli

**Figure 1:**
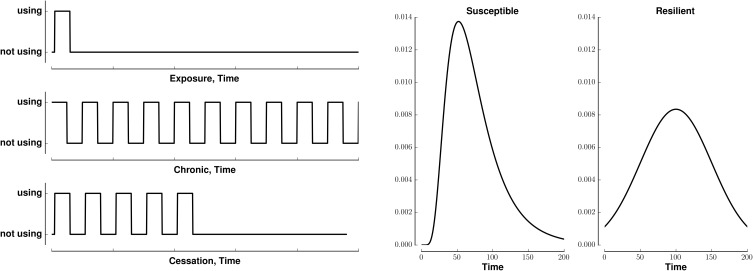
**Patterns of stimuli and rewards used as input. Left:** Templates for three different patterns of binary stimuli, isolated (exposure), tonic (chronic), and cessation. **Right:** Templates for two different dynamics of reward salience, log-Gaussian (susceptible) and Gaussian (resilient). All templates last for 200 time steps.

**Figure 2:**
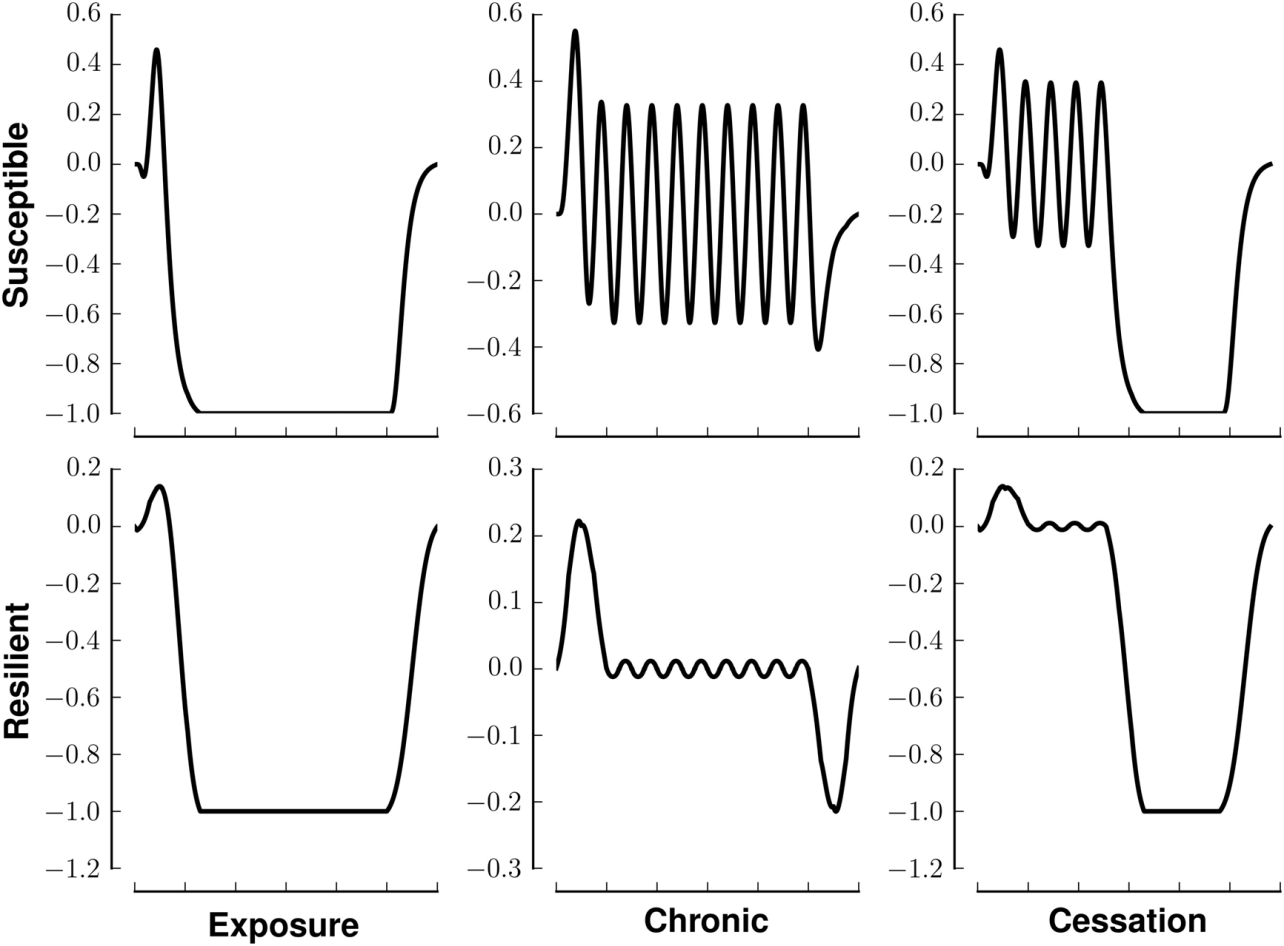
**Stimuli-reward patterns for simulation**. Each panel shows the reward in arbitrary units over time associated with different patterns of drug use. Rows denote different network modes, susceptible or resilient. Columns denote different patterns of drug usage, initiation (exposure), chronic (continual use), or cessation. All patterns last for 200 time steps.

We model (crudely) the initiation, continuation, and cessation of drug use with three patterns of stimuli, exposure, chronic, and cessation, respectively (Figure 1, left). We combine these stimuli with two types of reward saliences, designed to model susceptible and resilient individuals (Figure 1, right). The reward associated with a stimulus is a log-Gaussian for susceptible individuals and a Gaussian for resilient individuals. A log-Gaussian function was chosen to reflect experimentally observed dynamics of positive reinforcement (Koob and Le Moal, 2005; Koob, 2013). A Gaussian function was chosen to model the slower and softer dynamics suggested to occur in resilient individuals (Ersche et al., 2010). We calculate the stimulus-reward patterns as the convolution of each combination of stimulus and reward (Figure 2).

**Figure 3:**
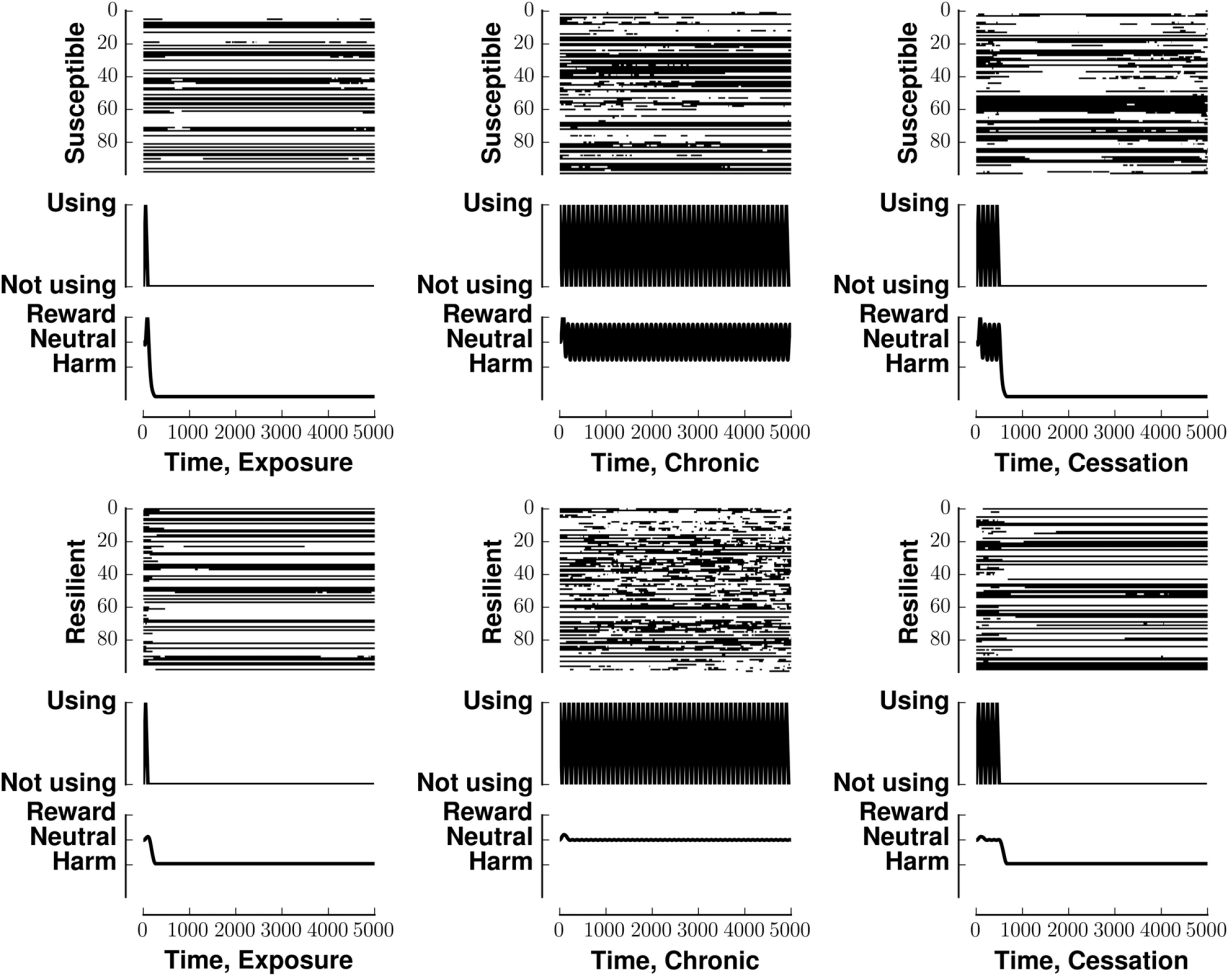
**Stability of network activity in the face of various stimulus-reward inputs**. Each panel shows the raster (top), stimulus (middle), and associated reward (bottom) for one of the six stimulus-reward patterns from Figure 2. The row (x-label of raster) indicates the reward pattern, susceptible or resilient. The column (y-label of raster) indicates the stimulus pattern (exposure, chronic, or susceptible). In the raster, each row indicates a neuron. The x-axis of the raster indicates time. A black mark is placed at the *it*th position if neuron *i* fired at time *t*. The simulations in all panels began with the same initial condition, being within the basin of attraction of **v**^0^.

Figure 3 investigates the ability of our network to maintain a preset pattern in the face of different stimuli and different rewards associated with those stimuli. In that figure, all panels in a row share the same reward. All panels in a column share the same stimulus. Each panel has three components, a raster plot, the stimulus, and the reward associated with that stimulus. The middle column, in which the stimulus is tonic, shows the greatest deviation from the resting pattern. Each row of the raster indicates the firing pattern of a neuron, with black indicating an action potential and white indicating the absence of firing. The middle graph in each panel indicates the stimulus pattern. The bottom graph in each panel indicates the perceived reward.

Figure 3 shows that susceptible networks are more able to maintain the preset pattern in the face of a chronic stimulus than resilient ones are; however, resilient networks can better maintain the present pattern once the stimulus stops. In the context of neural network computation, stability of our network in the face of different stimulus-reward patterns reflects (i) the incompatibility between the patterns the inputs would embed and the preset patterns embedded in the network, and (ii) the lower energy associated with the preset patterns which favors maintaining them. In the context of addiction, patterns that are stable in the face of input could model the lack of alteration of synaptic weights in resilient individuals or the perpetuation of destructive behaviors in susceptible individuals who develop substance dependence.

To quantify the similarity in patterns between two panels, we considered each of the *N* rows of each panel's raster to represent a vector. We calculated the similarity between two patterns, *a* and *b*, denoted by *q*_*ab*_, as the average of the cosine of the angle, θ, between each corresponding rows Equation (2).

(2) $$q_{ab}=\frac{1}{N}\sum_{n=1}^{N}\frac{v_{n,a}\;\cdot \;v_{n,b}}{\left||v_{n,a}||\;\cdot \;||v_{n,a}|\right|}$$

**Figure 4:**
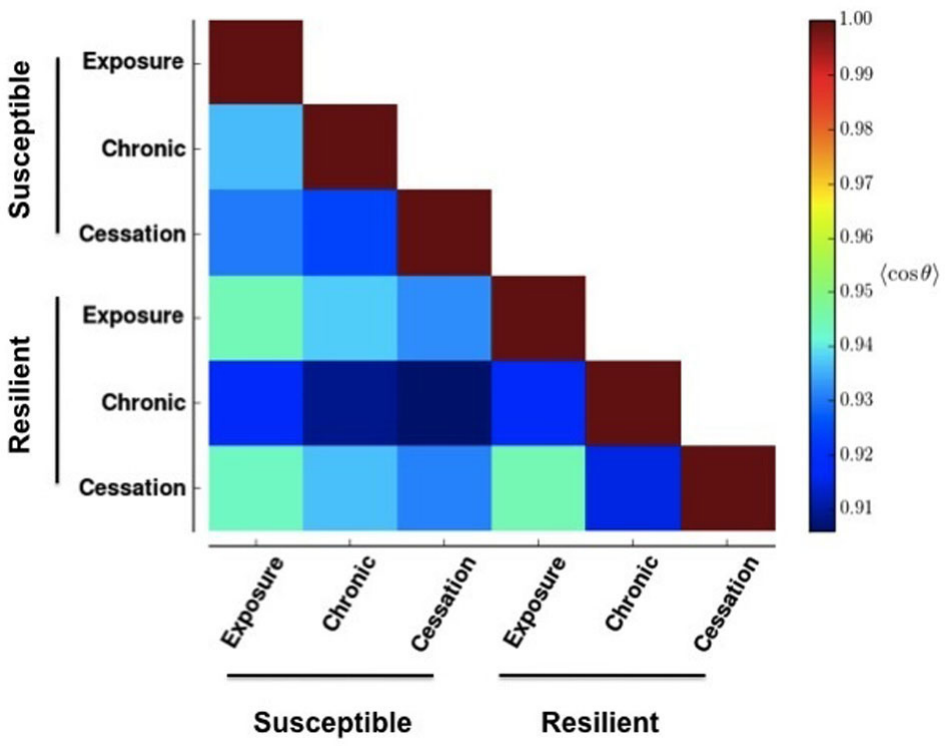
**Similarity between network activities in Figure 3**. The color of each box in the heat map shows the circular mean of the cosine of the angles formed between each row of the corresponding panels in Figure 3. A row makes an angle of 0 with itself, which corresponds to a cosine value of 1. Cooler colors indicate more different patterns.

Figure 4 shows the result of applying Equation (2) to Figure 2. Changes greater than this magnitude are beyond the 85^th^ percentile in the empiric cumulative distribution function created from randomly shufffling all rows in all rasters in Figure 3. This corresponds to a change in the cosine of the angle of more than 0.05. That is to say, the deeper blue the square, the more effective the stimulus-reward input was at embedding its pattern.

This stability (resistance to embedding) is lowest with the most prolonged stimulus, chronic use, as shown by the deep blue colors in Figure 4. The impairment persists only for networks whose reward correlation follows a susceptible scheme. In the lowest three rows in the first column of Figure 4, the square corresponding to chronic (prolonged) exposure is deep blue, but the others are paler than their counterparts in the susceptible scheme. Interestingly, the susceptible network has a more profound negative reaction than the resilient network does to initial exposure and sensation (bottom graph in the panels in Figure 3).

**Figure 5:**
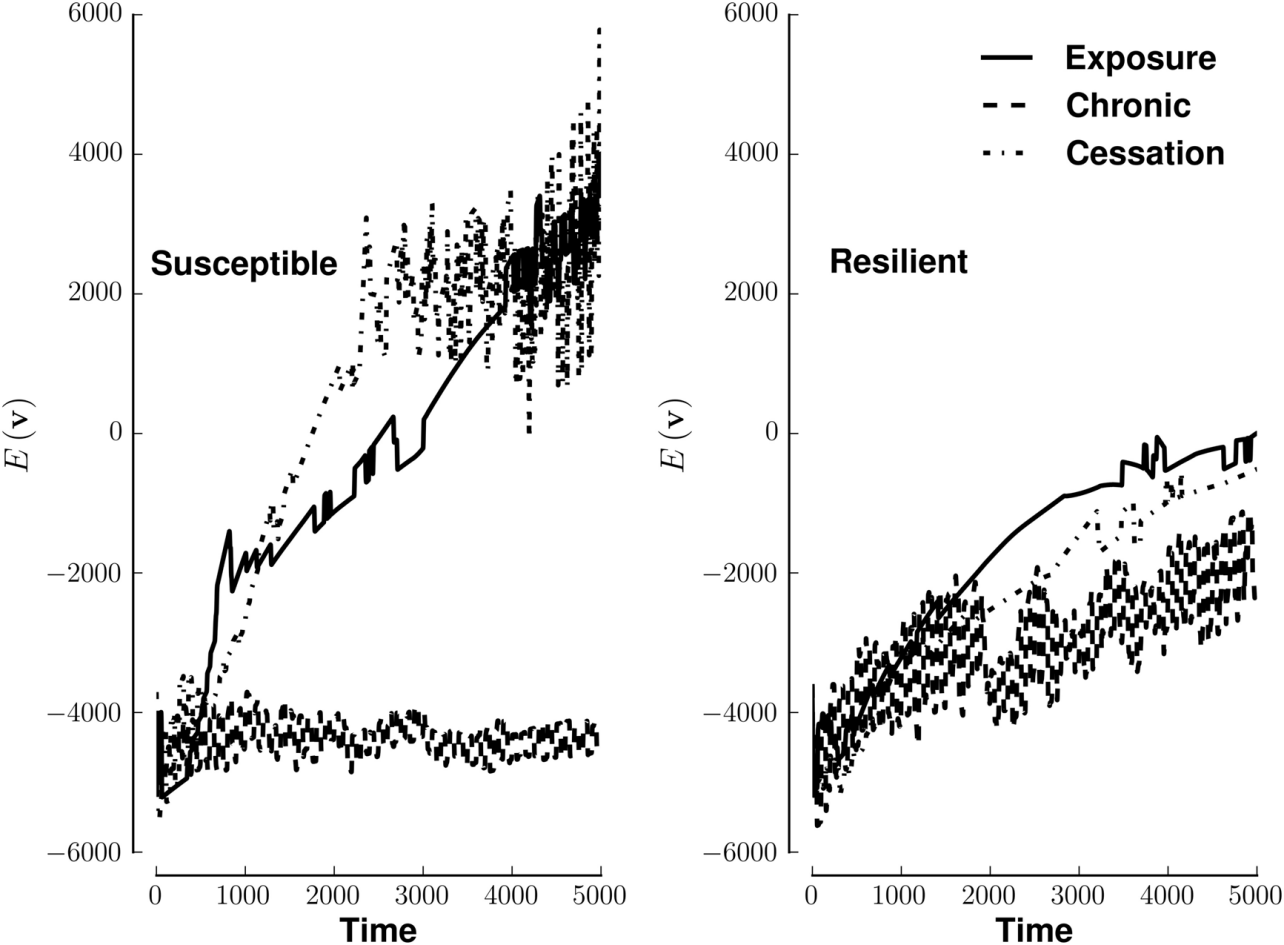
**Energy of network activity in the face of various stimulus-reward patterns**. Left panel shows the stability of a susceptible network when stimulated by exposure (solid line), continuous use (dashed line), or cessation (dashed-dotted line). Right panel shows similar conditions for a resilient network. The y-axis of each panel plots energy on the same arbitrary scale. See Methods for how *E*(**v**) quantifies stability. Lower energies are more stable.

**Figure 6:**
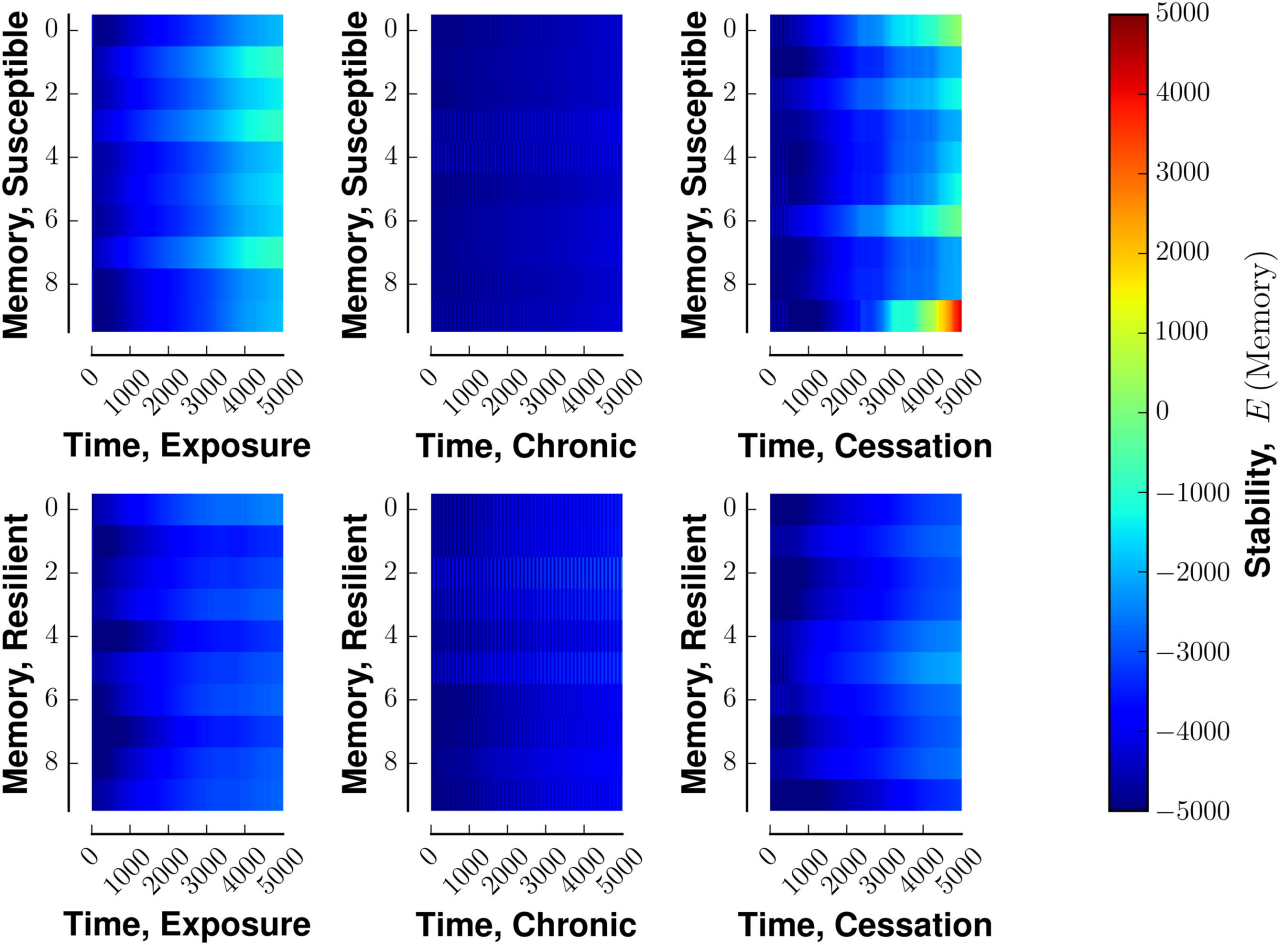
**Stability of fixed points of network in the face of various stimulus-reward schemata**. Layout similar to Figure 3. The top row denotes a susceptible reward profile, the bottom a resilient profile. The left column indicates exposure to rewarding stimulus, the middle column continuous use of a rewarding stimulus, and the right column shows the cessation of continuous use. Lower energies are more stable.

Susceptible networks exhibit more stable patterns of activity with continual exposure to a highly rewarding stimulus than do resilient networks (Figure 5). We calculated stability according to Equation (9) (see Materials and Methods). Taken with the impairment in recall, this suggests that, in susceptible networks, chronic use creates new fixed points while destabilizing existing ones. Figure 6 shows that previously stable patterns become associated with higher energies in susceptible but not resilient networks after ceasing to be exposed to a highly rewarding stimulus.

## 3. Discussion

This paper discussed the ability of a computational model of neural population dynamics with activity-dependent plasticity to maintain preset patterns of activity in the face of different stimulus-reward patterns. The types of stimuli were chosen to model patterns of drug use. Rewards and stimuli were chosen to reflect the division into susceptible and resilient organisms, noted in the experimental and clinical literature.

We found that a tonic stimulus, modeling chronic exposure, was most effective in destabilizing the network. If the network perceived rewards according to Gaussian (resilient) dynamics it fully recovered. If it perceived rewards according to log-Gaussian (susceptible) dynamics, then it remained altered. The discontinuation of the tonic stimulus promoted unstable network activity in networks that follows log-Gaussian (susceptible) but not Gaussian (resilient) reward dynamics. Our computational results agree with experimental and clinical findings. Chronic but not acute use causes cognitive impairment for many drugs of abuse (Block et al., 2002; Lundqvist, 2005). These impairments persist in some people even after cessation (Gouzoulis-Mayfrank et al., 2003). The chronic use of drugs of abuse impairs certain neurocognitive domains more than others (Bechara, 2005).

Simulating the relationship between synchrony and network activity may provide insight into the pathogenesis and treatment of functional brain disorders. It also suggests that certain patterns of deep brain stimulation may be more effective than others for a given pathology. For example, structured patterns of stimulation may be more effective for some neuropsychiatric disorders, while a noisier stimulus, similar to that used in electroconvulsive therapy, may be more appropriate for other disorders. In support of this postulate, the frequencies used in deep brain stimulation, even in the same region, vary with the disease being treated (McIntyre et al., 2004). Stimulation of the internal capsule and adjacent ventral striatum are effective for treating obsessive-compulsive disorder only at frequencies between 100 and 130 Hz (Greenberg et al., 2010). Tonic but not phasic stimulation of the medial prefrontal cortex at 100 Hz reverses a depressive phenotype in mice (Covington et al., 2010).

Future work, beyond addressing the caveats below, could investigate whether the stimulus-reward patterns used here induce similar effects in networks with different classes of embedded patterns. This network embedded patterns using a bivariate covariance rule. Many other schemes exist for embedding patterns, including those using multivariate covariance rules. Our model considered only the rewarding effects of drugs. A more realistic model could account for negative reinforcement of withdrawal, which may be more important in the maintenance of drug-seeking behavior (Koob, 2013).

### 3.1. Caveats

The network constructed here grossly simplified the interactions in neural networks, assuming that (i) all units in the network interacted linearly, (ii) the dynamics of the network followed a Markov chain, and (iii) there is no learning of new memories. These assumptions limit how widely the conclusions of this paper apply. The assumption of linear interactions simplifies the analysis. However, neuromodulators, such as dopamine and acetylcholine, are important in learning and memory and reward-dependent plasticity. Their effects on neural activity are non-linear. The Markov assumption simplifies simulation and allows the calculation of an energy function at the expense of making this network unable to manifest very slow correlations (Glauber, 1963; Kim and Nelson, 1999).

## 4. Materials and methods

### 4.1. Overview

This section details the construction of a model neural network with (i) excitatory and inhibitory connections, (ii) external input, and (iii) the ability to recover prior patterns of activity. For more detail, refer to the Supplementary Material. All computer code used in the project are available in the Github repository synchrony.

**Figure 7:**
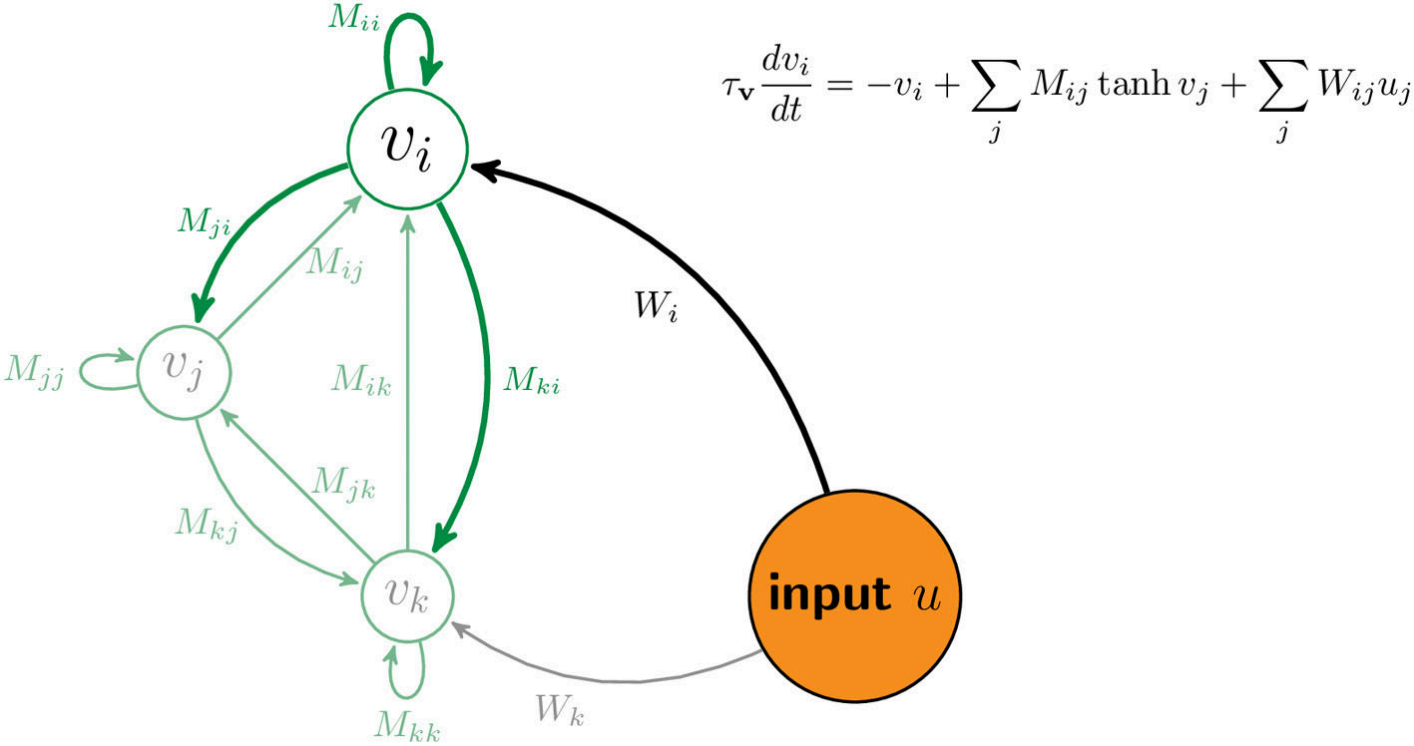
**Schematic of network**. The vector **v** denotes the firing rates of all neurons in the network. For illustration, the activity and connections of the *i*th neuron are highlighted while those of two other neurons are displayed but ghosted. The matrix element *M*_*ij*_ denotes the connection strength from the *j*th neuron to the *i*th neuron. The matrix element *W*_*ij*_ denotes the connection strength from *j*th component of the input, *u*_*j*_, to the *i*th neuron.

Figure 7 sketches a portion of the network with three neurons, *i*, *j*, and *k*. The matrix **M** contains the strength of connections between neurons. The matrix **W** contains the strength of connections between components of the input, **u**, and neurons in the network. Equation (3) describes the dynamics of the network. The equation inset in Figure 7 is a version of Equation (3) for one neuron.

(3) $$\tau_{v}\frac{dv}{dt}=-v+M\;\cdot \;F\left(v\right)+W\;\cdot \;u\;F\left(v\right)=\text{tanh }v$$

Equation (4) constructs a symmetric matrix, **M**, from a finite set of memories, {**a**^Ω^}.

(4) $$M=\frac{1}{\left(1-\alpha \right)\alpha |\left\{a^{\Omega }\right\}|}\sum_{\left\{a^{\Omega }\right\}}\left(a_{i}^{\Omega }-\alpha n\right)\left(a_{i}^{\Omega }-\alpha n\right)-\frac{1}{\alpha |\left\{a^{\Omega }\right\}|}$$

We introduce the terms Hebbian modification and anti-Hebbian modification to denote a strengthening or weakening of connections in the presence of correlated activity, respectively. Without an anti-Hebbian term in the dynamics of the recurrent connection matrix, **M**, each row of the feedforward weight matrix, **W** will come to lie parallel to the principal eigenvector of the input correlation matrix. This will make each target neuron respond identically. To break this redundancy we allow anti-Hebbian modification into the dynamics of **M**, using Equation (5) from Goodall (1960).

(5) $$\tau_{M}\frac{dM}{dt}=\left(I-M\right)-\left(W\;\cdot \;u\right)v$$

#### 4.1.1. Reward-dependent plasticity

Dysregulation of brain areas that process rewards plays a role in the pathogenesis of addictive disorders (Everitt and Robbins, 2005). A simple way to account for the rewarding effects of a stimulus, **u**, is to make the connections between that stimulus and the network, **W**, dependent on the magnitude of that reward, *r*. Equation (6) shows for one neuron, *v*, the Rescorla-Wagner rule, a simple mathematical formulation of this concept (Rescorla and Wagner, 1972).

(6) $$\begin{array}{l} \;v=w\;\cdot \;u\\ w\leftarrow w+\varepsilon \delta u\\ \;\delta =r-v \end{array}$$

In Equation (6), **u** denotes input to the network. The vector **w** weights those inputs. The scalar, ε, represents the associability of the stimulus with the reward. The vector, δ, denotes the reward-prediction error. This name for δ arises from interpreting the second line in Equation (6) as a gradient descent rule that minimizes the quantity 〈(*r* − *v*)^2^〉, which is the mean squared error between the actual reward, *r*, and the prediction, *v*.

Equation (7) modifies Equation (5) by incorporating Equation (6).

(7) $$\tau_{W}\frac{dW}{dt}=KWu\left(r-v\right)$$

#### 4.1.2. Stability of memories

If the state of any unit *i* in the network at some time *t* follows Equation (8), then network activity, **v**, evolves as a Markov chain (Glauber, 1963). Equation (8) assumes the activity of the *i*th unit, *v*_*i*_ follows Equation (3).

(8) $$P\left[v_{i}\left(t+\Delta t\right)=1\right]=\frac{1}{1+e^{-v_{i}}}$$

A network of binary units updated according to Equation (8) is often called a Boltzmann machine because once the network has reached equilibrium, a Boltzmann distribution defines the probability that a pattern of network activity will occur (Hinton and Sejnowski, 1986). In a classical Boltzmann machine, one unit is randomly selected and updated at each time point.

Every pattern of activity in the network, **v**, has an energy, *E*(**v**), associated with it [top line of Equation (9)]. Patterns with lower energy are more stable, that is more likely to occur, because they are more likely to occur. The probability of a pattern, **v** occurring, increases as the energy associcated with that pattern, *E*(**v**), decrease [bottom line of Equation (9)].

(9) $$\begin{array}{l} E\left(v,\;u\right)=-\left(u\;\cdot \;W\;\cdot \;v\right)+\frac{1}{2}\left(v\;\cdot \;M\;\cdot \;v\right)\\ \;\mathbb{P}[v]=\frac{e^{-E\left(v\right)}}{Z}\\ \;Z=\sum_{\left\{v\right\}}e^{-E\left(v\right)} \end{array}$$

### Conflict of interest statement

The authors declare that the research was conducted in the absence of any commercial or financial relationships that could be construed as a potential conflict of interest.
