# Supplementary material for: Synchrony can destabilize reward-sensitive networks
Source: Front Neural Circuits. 2014 Apr 30;8:44. doi: 10.3389/fncir.2014.00044 (PMC4012213; doi:10.3389/fncir.2014.00044)
Supplement: Supplementary file 1 [file DataSheet1.PDF]

MATHEMATICAL APPENDIX

REPRESENTING NETWORK ACTIVITY IN TERMS OF THE CONNECTION MATRIX

Equation (1) describes the projection of the network activity, $\mathbf{v}$, onto the vth eigenvector, $\mathbf{e}_v$. In Equation (1), $\mathbf{v}$ is an N-dimensional vector that denotes the firing rate of all N neurons in our network. The vector $\mathbf{h}$ denotes the input. The matrix, $\mathbf{M}$, denotes the connection strengths between units, that is M_{ij} denotes the strength of connection from the jth neuron to the ith one. The vector $\mathbf{e}_\mu$ denotes the μth eigenvector and λ_μ is its accompanying eigenvalue. The coefficient of projection, $c_v\,(t)$ measures the amount of network activity parallel to that eigenvector. It, unlike the vth eigenvector, λ_v, can vary with time because the network activity is itself a function of time.

$$\begin{aligned}
\tau_r \frac{d\mathbf{v}}{dt} &= -\mathbf{v} + \mathbf{h} + \mathbf{M}\cdot\mathbf{v} & \mathbf{v}\,(t) &= \sum_{\mu=1}^{N} c_\mu\,(t)\,\mathbf{e}_\mu. \\
\tau_r \sum_{\mu=1}^{N} \frac{dc_\mu}{dt}\mathbf{e}_\mu &= -\sum_{\mu=1}^{N}(1-\lambda_\mu)\,c_\mu\,(t)\,\mathbf{e}_\mu + \mathbf{h} & \mathbf{e}_\mu\cdot\mathbf{e}_v &= \delta_{\mu v}\text{ , pick } v\text{th one.} \\
\tau_r \frac{dc_v}{dt} &= -\,(1-\lambda_v)\,c_v\,(t) + \mathbf{e}_v\cdot\mathbf{h} & &\text{Assume } \mathbf{h}\neq\mathbf{h}\,(t) \\
c_v\,(t) &= \frac{\mathbf{e}_v\cdot\mathbf{h}}{1-\lambda_v}\left(1-e^{-\frac{t}{\tau_r}(1-\lambda_v)}\right) + \left(c_{v,\text{init}}\right)e^{-\frac{t}{\tau_r}(1-\lambda_v)}
\end{aligned} \tag{1}$$

Moving from the first line to the second line in Equation (1) used the expansion shown to the right of the first line. Moving from the second line to the third line uses the fact that eigenvectors are mutually orthogonal. We assume that the filtered input, $\mathbf{h} = \mathbf{W}\cdot\mathbf{u}$ is not a function of time so that we can easily separate the variables in second line for integration. The third line describes the projection of the voltage onto the vth eigenvector.

The final line in Equation (1) describes how the topology of a network constrains its dynamics by expanding the distribution of firing rates, $\mathbf{v}$, in terms of the eigenvectors of the connection matrix, $\mathbf{M}$, The third line in Equation (1) shows that the eigenvalue controls the scaling of the input $\mathbf{e}_v\cdot\mathbf{h}$.

$$\begin{aligned}
\tau_{\mathbf{M}}\frac{d\mathbf{M}}{dt} &= (\mathbf{I}-\mathbf{M}) - (\mathbf{W}\cdot\mathbf{u})\,\mathbf{v} & &\text{Analyze steady-state} \\
(\mathbf{I}-\mathbf{M}_\infty) &= (\mathbf{W}\cdot\mathbf{u})\,\mathbf{v} & &\text{Take tensor product with } \mathbf{v} \\
(\mathbf{I}-\mathbf{M}_\infty)\,\mathbf{v} &= (\mathbf{W}\cdot\mathbf{u})\,\mathbf{Q}_{\mathbf{vv}}
\end{aligned} \tag{2}$$

Equation (2) relates the autocorrelation of network activity, $\mathbf{Q}_{\mathbf{vv}}$, to the strength of recurrent and feedforward connections. Synchronous input can induce correlated network activity even if neurons in the network do no connect to each other, that is the left-hand side of Equation (2) does not vanish when $\mathbf{M}_\infty \to 0$.

Equation (3) shows how the basic Hebbian rule (**Hebb**, 1949) drives weights to lie parallel to the eigenvectors of the input autocorrelation matrix, $\mathbf{Q}$.

$$\begin{aligned} \tau_w \frac{d\mathbf{w}}{dt} &= v\mathbf{u} && \text{Substitute } v = \mathbf{w}\cdot\mathbf{u} \text{ because } \tau_r \frac{dv}{dt} = -v + \mathbf{w}\cdot\mathbf{u} \\ &= \mathbf{w}\cdot\mathbf{u}\mathbf{u} && \text{Define } \mathbf{Q} = \langle \mathbf{u}\mathbf{u} \rangle \\ &= \mathbf{Q}\cdot\mathbf{w} && \text{Recognize } \mathbf{Q} \text{ as correlation matrix} \end{aligned} \tag{3}$$

In Equation (3), $\mathbf{w}$ denotes the strength of connection between each neuron in $\mathbf{u}$ and v. The time constant for the adjustment of synaptic weights is represented by τ_w. Moving from the first to the second line assumes that the modification of synapses is much slower than synaptic transmission, which allows v to be replaced by its steady-state value. This is in keeping with the observation that synaptic plasticity involves protein synthesis, which is orders of magnitude slower than the movement of ions through a permeable channel. Any plasticity rule with the same form as Equation (3) ultimately sets synaptic weights parallel to the input autocorrelation matrix.

Equation (4) demonstrates that Equation (3) is unstable because the weights, $\mathbf{w}$, grow without bound .

$$\begin{aligned} \mathbf{w}\cdot\tau_w \frac{d\mathbf{w}}{dt} &= \mathbf{w}\cdot\mathbf{Q}\cdot\mathbf{w} \\ \tau_w \frac{d|\mathbf{w}|^2}{dt} &= \mathbf{w}\cdot\mathbf{Q}\cdot\mathbf{w} && \text{Recall } v_\infty = \mathbf{w}\cdot\mathbf{u}; \mathbf{Q} = \mathbf{u}\mathbf{u}^\dagger \\ &= v^2 \end{aligned} \tag{4}$$

The above discussion demonstrates the importance of synchrony in changing the possible dynamics of a system. Strongly correlated can change synaptic weights. These changes directly modulate one synapse and induce compensatory changes in others, to maintain homeostasis. However, certain patterns of activity can only occur or co-occur with certain distributions of weights. This suggests that a network can compensate for the destabilizing effects of only some types of correlated inputs but not others. One input could even interfere with our network adjusting its feedforward weights in response to another pattern. This resembles the phenomenon of ”blocking” in classical and operant conditioning.

RECOVERY OF A MEMORY

We define the recovery of a pattern of activity, $\mathbf{v}^\Omega$, as when the network's activity, $\mathbf{v}(t)$, puts the network within the basin of attraction of that fixed point so that the trajectory of the system approaches that fixed point, $\mathbf{v}(t) \to \mathbf{v}^\Omega$ for large t.

$$\mathbf{v}^\Omega = \mathbf{F}\left(\mathbf{M}\cdot\mathbf{v}^\Omega\right) \quad \text{Definition of fixed point} \tag{5}$$

In Equation (5), $\mathbf{M}$ denotes the connection matrix, as in prior equations. $\mathbf{F}$ denotes a function. The vector $\mathbf{v}^\Omega$ represents the memory being recalled. Two necessary and sufficient conditions (taken together) for the existence of fixed points are that (i) $\mathbf{F}$ saturates (**Grassberger and Procaccia**, 1983), and (ii) $\mathbf{M}$ is symmetric (**Dayan and Abbott**, 2001).

DYNAMICS OF INTRINSIC CONNECTIONS DECORRELATE OUTPUTS

$$\tau_{\mathbf{M}}\frac{d\mathbf{M}}{dt} = (\mathbf{I} - \mathbf{M}) - (\mathbf{W}\cdot\mathbf{u})\,\mathbf{v} \tag{6}$$

Equation (6) decorrelates the outputs, as Equation (7) shows by noting that Equation (6) pushes the network activity $\mathbf{v}$ towards a uniform distribution.

$$\begin{aligned}
\tau_{\mathbf{M}}\frac{d\mathbf{M}}{dt} &= (\mathbf{I} - \mathbf{M}) - (\mathbf{W}\cdot\mathbf{u})\,\mathbf{v} && \text{Recall } \mathbf{K} = (\mathbf{I} - \mathbf{M})^{-1}\text{; Find } \mathbf{K}_\infty \\
\mathbf{K}_\infty^{-1} &= (\mathbf{W}\cdot\mathbf{u})\,\mathbf{v} && \text{Multiply both sides by } \mathbf{K}_\infty \\
\mathbf{I} &= \left(\mathbf{K}_\infty^{-1}\cdot\mathbf{W}\cdot\mathbf{u}\right)\mathbf{v} \Rightarrow \mathbf{I} && \text{Recall } \mathbf{v} = \mathbf{K}\cdot\mathbf{W}\cdot\mathbf{u}
\end{aligned} \tag{7}$$

$$\begin{aligned}
\tau_{\mathbf{v}}\frac{d\mathbf{v}}{dt} &= -\mathbf{v} + \mathbf{W}\cdot\mathbf{u} + \mathbf{M}\cdot\mathbf{v} && \text{Notice } \mathbf{v}_\infty = \mathbf{W}\cdot\mathbf{u} + \mathbf{M}\cdot\mathbf{v}_\infty \\
\mathbf{K} &\equiv (\mathbf{I} - \mathbf{M})^{-1} && \text{Assume eigenvalues of } \mathbf{M} \text{ have Re } < 0 \\
\tau_{\mathbf{W}}\frac{d\mathbf{W}}{dt} &= \langle \mathbf{v}\mathbf{u}\rangle \Rightarrow \mathbf{K}\cdot\mathbf{W}\cdot\mathbf{Q}_{\mathbf{vu}} && \tau_{\mathbf{v}} \ll \tau_{\mathbf{W}}
\end{aligned} \tag{8}$$

In the last line of Equation (8), $\mathbf{Q}_{\mathbf{vu}}$ denotes the cross-correlation function between the stimulus, $\mathbf{u}$ and network activity, $\mathbf{v}$. Equation (8) is unstable because the right-hand side of the last line can grow without bound if the neurons in the network form functional connections with each other, that is $\mathbf{M} \neq \mathbf{I}$, or if they are sensitive to input, that is, not all elements of, $\mathbf{W}$ are 0. In the next section, we demonstrate how including the effects of reward-dependent plasticity provides one way to make Equation (8) stable.

CONSTRUCTION OF $\mathrm{M}_\dagger$

$$\mathbf{M} = (1 - \delta_{aa'}) \sum_{\{\mathbf{a}^\Omega\}} \mathbf{a}_i^\Omega \otimes \mathbf{a}_i^\Omega \tag{9}$$

Equation (10) derives $\mathrm{M}_\dagger$, a matrix that encodes the vectors $\begin{bmatrix} 1 \\ 1 \end{bmatrix}$ and $\begin{bmatrix} 0 \\ 1 \end{bmatrix}$ according to Equation (9).

$$\begin{aligned}
\mathbf{M} &= (1 - \delta_{aa'}) \sum_{\{\mathbf{a}^\Omega\}} \mathbf{a}_i^\Omega \otimes \mathbf{a}_i^\Omega \\
&= (1 - \delta_{aa'}) \left\{ \begin{bmatrix} 1 & 1 \\ 1 & 1 \end{bmatrix} + \begin{bmatrix} 0 & 0 \\ 0 & 1 \end{bmatrix} \right\} \\
&= \begin{bmatrix} 0 & 1 \\ 1 & 0 \end{bmatrix}
\end{aligned} \tag{10}$$

The last line of Equation (10) corresponds to $\mathrm{M}_\dagger$ presented in the text when $\alpha = 0$, that is when the rows are linearly independent.

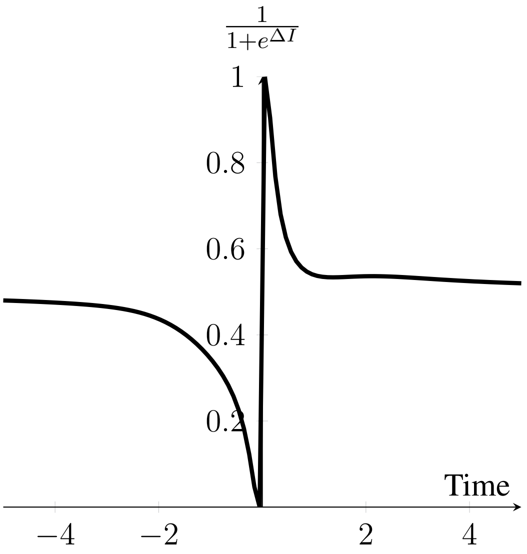

Figure 1. Difference in contributions of Gaussian and log-Gaussian reward dynamics to unit activity. Graph of Equation (12) assuming that both distributions have null mean, unit variance, and unit standard deviation.

COMPARISON OF REWARD DYNAMICS

In this section we analyze the effect that changing the temporal dynamics of the reward from a Gaussian to a log-Gaussian has on the chance that a neuron's state will change. We consider the activity of a neuron, I, in two states, Gaussian and log-Gaussian (Equation (11)). We assume that both distributions have null mean and unit variance and standard deviation.

$$\begin{aligned} I_{\text{Gaussian}} &= (\mathbf{M} - \mathbf{I})\,\mathbf{v} + k_{\text{Gaussian}} * s \\ I_{\text{log-Gaussian}} &= (\mathbf{M} - \mathbf{I})\,\mathbf{v} + k_{\text{log-Gaussian}} * s \end{aligned} \tag{11}$$

In Equation (11), s denotes a stimulus, which is the same for both cases. The symbol k denotes the Gaussian or log-Gaussian kernel, depending on the subscript. We now calculate the difference between the two lines of Equation (11) (Equation (12)).

$$\begin{aligned} \Delta I &= s * \left(k_{\text{Gaussian}} - k_{\text{log-Gaussian}}\right) \\ &= \frac{1}{\sqrt{2\pi}} \left(e^{\frac{-1}{2}t^2} - \frac{1}{t}\right) \end{aligned} \tag{12}$$

Figure 1 graphs Equation (12) to demonstrate that, given the same stimulus, reward dynamics that follow a Gaussian are more likely to change unit activity at the beginning of the stimulus. Reward dynamics that follow a log-Gaussian are more likely to change unit activity during the middle and at the end of the stimulus. When the two distributions have unequal variances or unequal means, Figure **??** is accordingly deformed.

COMPUTATIONAL APPENDIX

All code for this project was written by author in MC in Python and is available in the GitHub repository synchrony. The original data files are not on the repository because GitHub limits individual file size to 100 MB. The Python modules that recreate the figures in this paper, accordingly, will not run. The original data files are available on request.

NUMERICAL INTEGRATION

The basic integration scheme for all simulations was a modified forward Euler integration with a timestep of 0.01ms. For a timestep Δt, we used the update rule in Equation (13).

$$v_i\left(t+\Delta t\right) = v_{i,\infty} + \left(v_i\left(t\right) - v_{i,\infty}\right) + e^{-\frac{\Delta t}{\tau_r}} \tag{13}$$

In Equation (13), the steady state, v_∞ is given by Equation (14).

$$v_{i,\infty} = \frac{\mathbf{e}_i \cdot \mathbf{h}}{1-\lambda_i}\mathbf{e}_i \tag{14}$$

REFERENCES

Dayan, P. and Abbott, L. (2001), Theoretical Neuroscience, Computational Neuroscience (MIT Press)

Grassberger, P. and Procaccia, I. (1983), Characterization of strange attractors, Physical Review Letters, 50, 346

Hebb, D. (1949), Organization of Behavior (Wiley)
